# Choosing between virtual and in-person family physician care: a qualitative study

**DOI:** 10.1093/fampra/cmaf108

**Published:** 2026-01-09

**Authors:** Bridget L Ryan, Judith Belle Brown, Thomas R Freeman, Madelyn daSilva, Hazel Wilson, Rachelle Ashcroft, Amanda L Terry

**Affiliations:** Centre for Studies in Family Medicine, Western University, London, Ontario, Canada N6A 3K7; Department of Family Medicine, Western University, London, Ontario, Canada N6A 3K7; Department of Epidemiology and Biostatistics, Western University, London, Ontario, Canada N6A 3K7; Centre for Studies in Family Medicine, Western University, London, Ontario, Canada N6A 3K7; Department of Family Medicine, Western University, London, Ontario, Canada N6A 3K7; Centre for Studies in Family Medicine, Western University, London, Ontario, Canada N6A 3K7; Department of Family Medicine, Western University, London, Ontario, Canada N6A 3K7; Centre for Studies in Family Medicine, Western University, London, Ontario, Canada N6A 3K7; Department of Family Medicine, Western University, London, Ontario, Canada N6A 3K7; Centre for Studies in Family Medicine, Western University, London, Ontario, Canada N6A 3K7; Factor-Inwentash Faculty of Social Work, University of Toronto, Toronto, Ontario, Canada M5S 1V4; Centre for Studies in Family Medicine, Western University, London, Ontario, Canada N6A 3K7; Department of Family Medicine, Western University, London, Ontario, Canada N6A 3K7; Department of Epidemiology and Biostatistics, Western University, London, Ontario, Canada N6A 3K7; Schulich Interfaculty Program in Public Health, Western University, London, Ontario, Canada N6A 3K7

**Keywords:** family medicine, qualitative research, telemedicine, primary care, multi-morbidity

## Abstract

**Background:**

Virtual care accelerated to the forefront of family physician (FP) care following the COVID-19 pandemic and continues to play a significant role in patient care. The choice between virtual and in-person primary care must be sensitive to patients’ contexts particularly for those with multi-morbidity.

**Objectives:**

This study explored how to make the choice between virtual and in-person FP care for persons living with multi-morbidity that is acceptable to patients and FPs.

**Methods:**

We conducted a constructivist grounded theory study to understand the processes patients and FPs employ when deciding on the mode of primary care delivery. We used individual interviews to understand the perspectives and expectations of patients with multi-morbidity (2+ chronic conditions) and FPs.

**Results:**

There were two main themes revealed in data analysis: Considerations in choosing mode of delivery (including reason for visit, impact on access, technological logistics, and reimbursement for virtual care) and Process for choosing mode of delivery (including endorsing the patient choice when possible and scheduling visits).

**Conclusion:**

This paper integrated the experience of both patients and FPs to understand how to make the choice between virtual and in-person care. This understanding can support the future of FP care where diverse modes of delivery are employed, but currently technological barriers remain. Clinical scheduling systems that depend on telephone interactions between clinic staff and patients do not always support the process patients and FPs indicated they prefer; that is, one that respects patient preference and FP clinical expertise.

Key messages

- Four considerations when determining family physician visit mode of delivery
- Ideal process when choosing mode of delivery honors patient preference
- Booking systems unable to honor patient requests and family physician judgement
- Authors provide practical questions to choose between in-person and virtual care

## Introduction

Virtual care accelerated to the forefront of family physician (FP) care during the COVID-19 pandemic and continues to play a significant role in patient care [1]. Governing bodies in medicine are advocating for incorporating virtual modalities into primary care in ways that respect the patient-FP relationship, and ensure equitable access and continuity of care [2, 3]. The choice between virtual and in-person primary care must be sensitive to patients’ contexts, arguably being particularly important for those with poorer health and vulnerabilities [4]. Those living with multi-morbidity have complex health care needs, require frequent interactions with their FP [5], and experience high rates of social vulnerabilities [6–8] making accessing care difficult.

Patients are open to virtual care especially those who are employed, have mobility issues or mental health issues [9, 10]. The choice between virtual and in-person care also may have implications for providers if their choice of delivery mode affects their reimbursement. We are still in the relatively early stages of adaptation to virtual care as part of overall FP care; before the pandemic, virtual care accounted for a small portion of FP care [11]. We use the term virtual care to refer to both synchronous (e.g. video and telephone) and asynchronous (e.g. E-mail) modes of care. Understanding the complexities of adaptation to the integration of technologies in a complex healthcare system can be challenging [12]. We must address these complexities by examining the perspectives and expectations of both patients and FPs [13]. This current study aimed to explore how to make the choice between virtual and in-person FP care for persons living with multi-morbidity that is acceptable to patients and FPs.

## Methods

### Study design and participants

We conducted a constructivist grounded theory study [14] (CGT) to understand the processes patients and FPs employ when deciding on the mode of primary care delivery, including both virtual and in-person care. We used individual interviews to understand the perspectives and expectations of patients and FPs. We recruited: (i) patients who self-identified as having multi-morbidity (two plus chronic conditions) and had received virtual care from their FP; and (ii) FPs who had provided virtual care. We recruited patients and FPs independently from each other; that is, we did not recruit patient-FP dyads. We aimed for participant variation in age, gender, urban and rural locations, health status (patient), years in practice (FP), and payment/practice model (FP). Our interdisciplinary research team consisted of researchers, physicians, and a patient partner.

Patient and FP participants were recruited through several channels including social media advertisements, printed posters, and reaching out to Department of Family Medicine colleagues for recruitment assistance. Informed consent was received from all participants prior to data collection and confidentially was assured before starting the interview.

### Study context

Our study took place in Ontario, Canada. At the start of the pandemic, the Ontario government through the Ministry of Health established temporary billing codes for virtual care whereby both telephone and video visits were reimbursed at the same rate as in-person visits; these codes were in effect until November 2022 [15]. As of December 2022, the government agreed to a new virtual care funding framework with the Ontario Medical Association, which negotiates changes with the government [15]. One significant change was that comprehensive virtual care services rendered by video are payable at fees equivalent to in-person fees; however, most services rendered by telephone are payable at 85% [16]. The government published a bulletin on 1 September 2022 that made FPs aware that the framework was changing, but this bulletin did not provide specific details [17]. Our data collection occurred under the old and new virtual care frameworks.

### Data collection

Individual semi-structured interviews were conducted with participants via Zoom or telephone by a research team member (B.L.R. and Md.S.) from February 2022 to May 2023. For patients, five interviews were conducted prior to 1 December 2022 (prior to the new virtual care framework) and the remaining 14 were conducted after 1 December 2022. For FPs, eight interviews took place before 1 September 2022 with the remaining six conducted between September 1 and 30 November 2022 when FPs could have been aware of the new virtual care framework. We asked about participant experiences with virtual and in-person FP care, defining virtual care as both synchronous and asynchronous modes of delivery. Interviews were audio-recorded and transcribed verbatim. Data were managed using NVivo 20 software. Data collection and analysis were iterative and interviews were conducted until authors determined that data sufficiency was reached. Data sufficiency is determined when no new themes emerge from the data [18–21]. Having reached data sufficiency, the authors had confidence in their interpretation of the data [18–21].

### Data analysis

Consistent with the CGT process, our analysis consisted of line-by-line, focused and theoretical coding [14]. Open coding was conducted independently by four research team members (B.L.R., J.B.B., T.R.F., and Md.S.). The research team members met frequently to discuss and develop axial codes by grouping open codes. In consolidating the codes, we used a constant comparative analysis examining the data within and across interviews [14] thereby supporting the main themes. The final analysis stage, theoretical coding, examined connections and differences among the major themes and identification of quotes reflecting each theme and sub-theme [14].

Trustworthiness and credibility of the analysis were ensured by audio-recording interviews with verbatim transcription, and independent and team analysis. Researchers engaged in reflexivity throughout by discussing their personal and professional biases as disclosed in Table 1 [22, 23]. The online supplementary material provides the Standards for Reporting Qualitative Research (SRQR) statement.

**Table 1:** Declaration of biases for the seven authors consistent with CGT methodology (2022–25).

Author	Declaration
Bridget L. Ryan	B.L.R. is a PhD epidemiologist with mixed methods primary care research.
Judith B. Brown	J.B.B. is a PhD in social work with qualitative research expertise.
Thomas R. Freeman	T.R.F. is a FP and researcher with qualitative research expertise.
Madelyn da Silva	M.dS. holds an MSc in health and rehabilitation sciences and has experience with qualitative primary care research.
Hazel Wilson	H.W. is a patient and caregiver who have been involved in patient advocacy for several years. She has been heavily involved in patient partners in arthritis as well as research papers and conferences.
Rachelle Ashcroft	R.A. is a PhD in social work with qualitative expertise in primary care research.
Amanda L. Terry	A.L.T. holds a PhD in epidemiology and has experience with quantitative and qualitative research in family medicine and primary care.

Ethics approval was received from The University of Western Ontario Health Sciences Research Ethics Board (REB#120814).

## Results

Interviews were conducted with 19 patients (average 40 minutes) and 14 FP participants (average 50 minutes). Table 2 describes participant demographics. The majority (58%) of patient participants lived in one large urban center with the remaining from other locations across the province including both rural and urban areas; FP participants represented the geographical areas of Ontario but the majority was in urban settings. FPs most commonly worked in capitated practice models where physicians are compensated primarily through capitation payments based on rostered patients, but also receive fee for service payments and enrolment-specific bonuses and premiums [24].

**Table 2:** Characteristics of 19 patient and 14 FP participants (2022–23).

Characteristic	Patients (*n* = 19)	FPs (*n* = 14)
Age (x̄, SD)	66.4, 16.2	47.9, 13.3
Gender (*n*, %)		
Male	3, 16%	5, 36%
Female	15, 79%	9, 64%
Non-binary	1, 5%	0, 0%
Race (*n*)		
Caucasian	16	11
Other	3	7
Self-reported health conditions (*n*)		
Arthritis	13	N/A
Cancer	4	N/A
Diabetes	5	N/A
Gastrointestinal	3	N/A
Hypertension	4	N/A
Mental health	7	N/A
Musculoskeletal conditions	9	N/A
Other	18	N/A
Location (*n*)		
Urban	16, 84%	12, 86%
Small town/rural	3, 16%	2, 14%

### Overall themes

There were two main themes revealed in data analysis: Considerations in choosing mode of delivery and Process for choosing mode of delivery. While we asked about both synchronous and asynchronous modes of FP care delivery, participants spoke almost exclusively about synchronous modes (in-person, telephone, and video visits) with an occasional participant mentioning E-mail.

### Considerations in choosing mode of delivery

The first theme highlighted the considerations that need to be addressed to ensure the mode of delivery is appropriate for the visit; these considerations include four sub-themes: “reason for visit, impact on access, technological logistics and reimbursement for virtual care.” We grouped these considerations into their own theme rather than including them in the second theme concerning the process for choosing model of delivery, despite the inter-connectedness between the two themes. We did so because different considerations may inform different parts of the process.

#### Reason for the visit

This sub-theme highlighted the importance of considering the reason for the visit in deciding the appropriate mode of delivery.

Always clinical judgement needs to play a role. If it needs to be in-person it needs to be in-person, but a lot of things, as we’ve kinda come to know and surprisingly, not everything has to be handled, necessarily, in-person, like we thought. (FP3)

Often virtual visits were characterized as being appropriate “when it's a discussion issue, not an examination issue” (Pt 15).

#### Impact on access

An important consideration in choosing mode of delivery was virtual care's ability to enhance access; “In a well-designed system, I think it [virtual care] absolutely improves access. No doubt about it” (FP5). Participants elaborated practical reasons that virtual care was beneficial to them, “when I was working I’d have to take an hour or two off to go to the doctor” (Pt 13). For some participants, their degree of multi-morbidity meant that in-person visits created significant physical hardship.

If I'm really not feeling well, I don’t have to go through that physical hardship… you don’t have to go through the whole process of getting there. So, it's lovely to be able to… just speak to the doctor [virtually]. (Pt5)

#### Technological logistics

Technological considerations played a role in determining if a mode of delivery would be possible/practical. This included internet bandwidth, “I don't have high speed internet where I live. So I don't think having a face-to-face virtual is even possible. I wouldn't even bother trying” (Pt 4). Additionally, there were considerations regarding availability and comfort with the technology.

My patients very quickly showed me that, you know, video calls isn’t really going to be feasible with a lot of the elderly people… a lot of them don’t have a computer, don’t know how to work one, don’t have an email address that they check regularly. (FP3)

#### Reimbursement for virtual care

This sub-theme considered the role that reimbursement plays in making virtual care a viable choice in mode of delivery. Participants indicated that providing reimbursement for virtual care was a benefit that came out of the pandemic.

There's many situations where things could have been resolved [pre-pandemic] with a phone call. But because it wasn't offered, it wasn't financially offered or supported technologically by the government. It wasn't even an option. I think that's one of the good things that's come out of the pandemic, that we now use the phone and accept it, and reimburse physicians for it. (Pt4)

Participants also indicated that they thought it was important that reimbursement for virtual care continue post-pandemic, “It would be hugely backwards for the government to put more rules on restricting virtual access [in this context, participant was referring to reimbursement], for those of us that do believe that it's best to have the hybrid models” (FP9). Another participant stated, “payment is obviously a big issue…if a doctor is going to be using the telephone…they should be compensated much the same as they would be for face-to-face as there should be no difference” (Pt8).

### Process for choosing the mode of delivery

#### Endorsing patient preference when possible

Moving from “considerations in choosing” the mode of delivery to the “process for choosing” mode of delivery, overwhelmingly patient and FP participants expressed that this decision should rest with the patient when possible.

My job is to provide you with all of the information and help you to weigh the risks versus benefits of the alternatives and then it's your job to make the decisions … I would present them with the information and say this is what I suggest for you. I know which patients are working, I know which ones are trying to juggle three kids with after school activities and then driving over to my office where it's going to be an increased burden. (FP4)

Sometimes the patient's choice of mode of delivery was made within the context of the patient-FP relationship with participants recommending there be an explicit conversation about choice.

I think that should be a conversation between the patient and the family doctor, at some point. What are your preferences? What are your reasons for those preferences? …Some people prefer to be seen in-person all the time. And I think that should be respected. I think my doctor and I just kind of have an understanding, the phone works for me, it works for her. And we’ll just carry on. (Pt4)

However, this was rare with some participants indicating that this kind of conversation between patients and their FPs is not happening, “I don’t think we’re doing a really good job of giving patients and families choice and deciding when these visits have to happen in-person versus when they can happen over video” (FP1).

Further, some participants indicated that, post-pandemic, there seemed to be a shift away from patients being able to choose virtual care, “[Virtual care] hasn’t been offered, and I haven’t heard any sort of suggestion that it might be… I think they’re moving more towards the in-person, and they’re not going to be doing virtual like they were” (Pt3).

Within the process of patients choosing the mode of delivery, the considerations from Theme 1 (such as “reason for visit”) informed, and could even dictate, the patient's choice.

Patients have the option of having virtual versus in-person—they should be able to have that option. The doctor should be OK with that. I mean that—in an ideal world. But also the doctor has to be able to bring the patient in when they need to see them. (Pt13)

Occasionally, when a patient chose a virtual visit, their FP would decide during that visit that a subsequent in-person visit was necessary, “Some of those phone calls still resulted in, ‘OK, well I still do need to see you in person’, right. But some [phone calls] didn’t. Like, a huge bulk of what we do as family doctors is verbal” (FP12).

#### Scheduling visits

Scheduling an appointment with their FP often occurred with the patient calling the FP's clinic and speaking with a staff member. This process usually resulted in staff making the choice on mode of delivery yet was occasionally determined through a discussion and mutual decision-making process between the patients and clinic staff.

I think the model that our Family Health Clinic uses… [is] me calling and them screening me to a certain extent. Because they’re quite competent. I’ve never felt that they were putting me off or steering me any way. It's been a mutual decision [on mode of delivery] and I like that and I think it works. (Pt8)

FPs also described the role of the clinic staff in choosing the mode of delivery, with the staff considering the clinical needs within their scope of practice, “I think our receptionists do a very good job at triaging in terms of knowing what may be more appropriate to be seen in-person. And we've also given them a list of things [that can be done virtually]” (FP 14).

While most patient participants talked about calling the clinic to schedule an appointment, some patient participants described having access to an online booking system, in which case they could choose the mode of delivery when scheduling an appointment, “They have an online booking system which I think is great, you can pick your time. So you do that and then they have to approve [the mode of delivery] and they get back to say it's set” (Pt3).

As well as scheduling an appointment by telephone or online, participants noted that sometimes scheduling a next appointment happened during the patient-FP encounter. This allowed the patient and the FP to decide together on the appropriate timing and mode of the next visit, “If I’m on the phone with someone and they require a follow-up visit, I can book the visit for them sometimes as well which sometimes is easier if I know exactly when I want [to] get them in [next] time” (FP4).

This was considered efficient when patients needed to be seen routinely, as is the case for those with multi-morbidity, “Whereas maybe because I've a higher proportion of people with chronic issues or mental illness, we're doing a lot of—‘I'd like to see you in three months or six months, and here is your next appointment’” (FP11).

## Discussion

Our findings highlight the path forward for choosing between in-person and virtual care for FP visits. We identified two main themes from the data. The first theme highlighted considerations to be addressed to ensure the mode is appropriate for the visit; these considerations included “reason for visit, impact on access, technological logistics and reimbursement for virtual care.” The second theme described the process for choosing the mode of visit, with sub-themes focused on “endorsing the patient choice when possible and scheduling visits.” Our findings demonstrate the interconnected and interrelated nature of our themes; the considerations discussed in Theme 1 cannot be separated from the process of choosing described in Theme 2. Additionally, the considerations and process are not fixed for every patient at every visit, but rather can vary by person and by need.

Theme 1 highlighted the considerations that our participants identified as being important when determining the best mode of delivery for a particular patient at a specific appointment. Consistent with other literature [25–28], our findings highlighted considerations such as the “reason for visit” and the ability of virtual care to “positively impact access” (e.g. removing travel time when an appointment is virtual) that plays a role in determining the best mode of delivery. These considerations can have greater impact on vulnerable patients [29] such as the patient participants we interviewed who had multi-morbidity. The “technological logistical” consideration in choosing mode of delivery such as lack of internet bandwidth has been noted in other pandemic-era literature [13, 30, 31]. Finally, consistent with other research [13, 32, 33], participants discussed the role of “reimbursement for virtual care” in the choice of mode of delivery. While participants valued virtual care and endorsed that choosing mode of delivery should primarily rest with the patient, they wondered if, in future, virtual care may no longer be reimbursed, and therefore may no longer be an option. Our data were collected during a timeframe when Ontario experienced a change in the virtual care funding framework where the Ministry of Health discounted the remuneration for telephone visits compared to in-person and video visits [16]. Although 14 patient participants were interviewed after the change in the funding framework, we do not know if they were aware of the discounting of telephone visits and whether that precipitated their worry. For the six FP participants interviewed from September to November 2022, they would have been aware of the upcoming virtual care framework change but perhaps not that it included the discounting of telephone visits. Therefore, we cannot ascertain from our findings if participants’ concerns were related to the policy change. Future research should evaluate if in fact a policy change discounting telephone visits leads to less virtual care.

Theme 2 encompassed the actual process of scheduling visits and included two sub-themes. The first sub-theme involved “endorsing patient preference” when the considerations (from Theme 1) support the ability to do so. Participants described an ideal process where patients and their FPs would have an initial conversation to decide on mode of delivery by discussing each other's considerations and preferences. Clinics and FPs may wish to consider adopting this process as standard practice. This is consistent with Stewart et al. that emphasizes the importance of finding common ground between a patient and their FP concerning treatment and management plans, which is a central component of patient-centered care [34]. The second sub-theme described the different ways participants reported “scheduling visits.” This second sub-theme is in tension with the first; while the majority of patient and FP participants emphasized the importance of listening to patient preference, participants also expressed that this is often not how the process unfolds in practice.

Themes 1 and 2 reflect the complex process of decision-making around virtual care provision.

As with most health technologies, classic socio-technical considerations come into play with virtual care adoption [12]. Workflow and organizational issues for example can impact the uptake of technology-based interventions in addition to technical considerations such as accessibility of hardware and software [35]. In this study, our participants expressed the ideal process for choosing between virtual and in-person FP care was to honor patient preferences. However, traditional clinical scheduling systems that depend on telephone interactions between clinic staff and patients are not designed to easily honor the patient's request while also considering the clinical judgement and clinic organization of the FP. Some clinics provided sufficient guidance to their clinic staff to allow them to triage to in-person or virtual visits facilitating patient choice. In the future, online booking systems may provide a further way to promote patient preference. External pressures operate within health technologies [35], and we identified this as one of the concerns FPs expressed about remuneration for virtual care.

**Figure 1:**
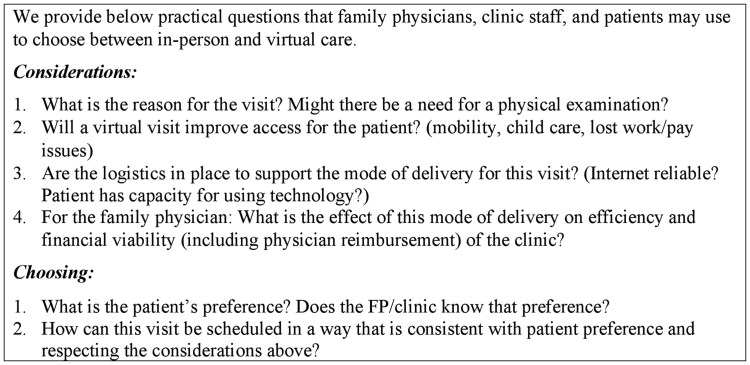
Questions to guide the choice between in-person and virtual FP care.

Our findings are consistent with viewing the patient/physician dyad as a complex adaptive system [36], and its resilience, when challenged by sudden changes in context, lies in the strength of the relationship. While our study took place in Ontario, Canada, we believe this complex adaptive nature expands to other healthcare contexts including the United States, Britain and Europe. Having reflected on the study findings, we developed a set of practical questions that can support the patient-FP relationship in addressing the considerations and processes we identified. In Fig. 1, we provide practical questions that FPs, clinic staff, and patients may use to choose between in-person and virtual care. While our population of interest was older adults with multi-morbidity, the nature of our findings, and the practical questions arising from them, have broader implications and may be useful for the general family practice population, being equally suited to episodic, health promotion and preventive care visits.

### Strengths and limitations

This paper adds to the literature by using CGT to compare and contrast perspectives of patients and FPs concerning how to choose between in-person and virtual FP care. The study was limited to those living with multi-morbidity; therefore, it may not be applicable to a general patient population. Our patient participants were mostly from one urban center and so the patient perspectives of those in smaller urban and rural areas may not have been adequately reflected. Our FP participants represented the broader geography of Ontario but were still concentrated in urban areas. We know that there are particular connectivity problems for those in smaller cities and rural areas. We cannot discern from this study whether this connectivity issue would have any impact on how patients and FPs chose between virtual and in-person care.

## Conclusion

Our study explored virtual care in Ontario, Canada; however, these findings can be applied to family medicine practices internationally. This paper integrates the experience of both patients and FPs to understand how to make the choice between virtual and in-person care. This understanding can support the future of FP care where diverse modes of delivery are employed, but currently technological barriers remain. Clinical scheduling systems that depend on telephone interactions between clinic staff and patients do not always support the process patients and FPs indicated they prefer; that is, one that respects patient preference and FP clinical expertise.

## Supplementary Material

cmaf108_Supplementary_Data

## Data Availability

Data cannot be made available according to our REB approval and the potential of qualitative data to identify participants.
